# Improvement plans on the operation of the Institutional Animal Care and Use Committee: focusing on the case of Seoul National University

**DOI:** 10.1186/s42826-022-00137-0

**Published:** 2022-08-18

**Authors:** Na Ahn, Jaehak Park, Jungjoon Ihm, Sangho Roh

**Affiliations:** 1 Laboratory Animal Medicine, College of Veterinary Medicine, Seoul National University, Seoul, 08826 Korea; 2 School of Dentistry and Dental Research Institute, Seoul National University, Seoul, 08826 Korea

**Keywords:** Animal ethics, Animal welfare, Education, IACUC, Laboratory animals

## Abstract

**Background:**

The Institutional Animal Care and Use Committee (IACUC) became compulsory in 2008 by the Animal Protection Act in Korea. Seoul National University (SNU), which conducts 5% of Korea’s total animal protocol reviews and uses 10% of national laboratory animal usage, has been influential in the review of animal protocols and management of animal facilities. This study was undertaken to suggest the operational improvement of the IACUC. It focused on the case of SNU.

**Results:**

The methodological framework consists of a qualitative approach. In particular, this study is focused on the grounded theory approach and sixty people were surveyed through purposeful sampling. Through this study, we found that various practical educations are necessary such as: (1) education for researchers on how to write a protocol, (2) standardization of screening criteria for various animal experiments by presenting various cases, (3) training on a detailed understanding of relevant laws and policies. In particular, an integrated management system, making it possible to share information among the related committees, would be essential for smoother operation of the IACUC.

**Conclusions:**

If various levels of education and the integrated management system are established, it will be possible to enhance the excellence of researchers and to better manage the operation of the IACUC.

## Background

The Institutional Animal Care and Use Committee (IACUC) became compulsory in 2008 by the Animal Protection Act in Korea [1]. In particular, Seoul National University (SNU) first launched the Animal Experiment Committee (SNU-AEC) in 2005, and the SNU-AEC changed its name to the IACUC in 2008, following a government mandate [2]. Subsequently, SNU has been influential in the review of animal protocols and management of animal facilities. However, the administrative systems in SNU are divided into two sections: Research Affairs supporting the operation of the IACUC, and the Institute of Laboratory Animal Research (ILAR) managing the animal facilities. However, the governmental regulations related to animal experimentation are also controlled by two laws: the Animal Protection Act (APA) and the Laboratory Animal Act (LAA) [1]. Thus, there are overlapping areas and blind spots in the management system for animal experimentation.

As animal experiments with genetically modified organisms and human materials gradually increase in research, communication with relevant committees such as the Institutional Review Board (IRB) and Institutional Biosafety Committee (IBC) is becoming essential. However, as the government departments managing these committees differ, the researchers, confused by complex regulations and guides, have raised dissatisfaction with the IACUC and the university. This is an issue faced by animal researchers, committee staff, and animal facility managers. Moreover, since the IACUC is an “institutional committee,” if it meets its purpose and values, its way of operation significantly varies between institutions and countries. A number of studies have analyzed the status of animal experiments and related guidelines [1, 3, 4]; however, there has been limited research except a review paper reporting from the establishment of an institution IACUC to its current status in Korea [2]. To our best knowledge, there are limited studies that investigate and analyze problems and improvement measures in the operation of IACUC.

Grounded theory is often used to explain specific cases or provide a study framework. Grounded theory is a qualitative research methodology that explores practical fields in order to explain specific social phenomena or obtain a new understanding of existing social phenomena. Therefore, grounded theory is a qualitative research methodology useful in understanding the social, psychological, and structural phenomena of humans and societies that are difficult to capture through other research methodologies [5, 6]. Grounded theory provides researchers and readers with understanding about specific social phenomena and provides meaningful insight into human action and interaction by strengthening their understanding [7]. Therefore, grounded theory attempts abstract conceptualization and theorization of the process and trajectory surrounding human behavior and interaction, thereby developing both the substantive and formal theories of social reality [8]. Grounded theory methodology has become the most representative qualitative research methodology in social science fields such as education, nursing, business administration, family studies, gerontology, social work, women’s studies, and cultural research [9–11]. In addition, the coding method of the grounded theory method is widely used as a representative analysis technique in qualitative research.

This study was conducted by collecting opinions from related parties inside and outside the school regarding the operation of the IACUC at SNU (SNU-IACUC), which leads the animal experimentation-related field at national level and has an overwhelming scale in terms of quantity and analyzed respondents’ opinions using the grounded theory methodology. The study was also undertaken to suggest the operational improvement of the IACUC and other relevant committees, in addition to related organizations. This is useful information for animal experimenters, researchers, practitioners in the SNU and Korea, and related parties globally.

## Results

### Demographic characteristics of the respondents

The frequency and proportion of participants according to their majors, careers, and their roles within the IACUC are presented in Table 1. Many of the survey respondents had majored in veterinary and animal sciences because both the principal investigator and researchers, and also a significant number of animal facility operators and the IACUC members and administrators, had majored in the relevant fields. Since the College of Medicine has animal facilities on three campuses (Yeongeon, Pyeongchang, and Hongcheon) within SNU, and the College of Pharmacy also operates large-scale animal facilities in Gwanak campus to conduct animal research, the ratio of principal investigators and researchers who majored in these two colleges is second highest after veterinary and animal sciences. Consequently, the survey request to the researchers was calculated at an appropriate rate.

**Table 1:** Demographic characteristics of survey participants

Items	Classification	Frequency (%)	Remarks
Roles in animal experiment-related activities	Committee member (= protocol reviewer) (CP)	19 (31.7)	Internal member: 12 External member: 7
	Administrator (AD)	7 (11.7)	
	Facility operator (FO)*	7 (11.7)	
	Principal investigator (PI)*	15 (25.0)	
	Postdoc or student (PS)*	12 (20.0)	
Specialty	Natural sciences (biology, chemistry, ecology, etc.)	10 (16.7)	
	Medical and pharmaceutical sciences	17 (28.3)	
	Veterinary Sciences	21 (35.0)	
	Others (Engineering, education, humanities, theology, etc.)	8 (13.3)	
	Unverifiable	4 (6.7)	
Affiliation	Seoul National University	45 (75.0)	
	Other universities	8 (13.3)	Public: 4; Private: 4
	Institution other than university	7 (11.7)	Public: 4; Private: 3
Experience of animal experiment-related activities	Less than 1 year	2 (3.3)	AD: 2
	1–10 years	19 (31.7)	CP: 4, AD: 1 FO: 2, PS: 12
	Over 10 years	36 (60.0)	CP: 15, AD: 3 FO: 3, PI: 15
	Unverifiable	3 (5.0)	AD: 1, FO: 2

*Surveys on FO, PI and PS were performed only to participants of Seoul National University

As a result of examining the period during which the respondents conducted activities related to animal experiments such as research, facility management, protocol review, committee administration, etc., most of the reviewers and the principal investigators were classified as having over 10 years of experience in animal experiment-related experiences. If the respondents had performed research for over 10 years as a researcher and acted as a reviewer for the last five years, they were classified as those with over 10 years of experience in relation to animal experiments.

### Open coding

All responses to the questions were summarized, while similar answers from the raw data were grouped into one item for the questionnaires voluntarily described by the respondents. Subsequently, the response rate for each item was summarized in the tables below.

#### Status of animal protocol review

The results of a questionnaire surveyed to the administrators of each institution’s IACUC about the scale of each institution’s animal protocol reviews are shown in Table 2. Excluding one private company and SNU-IACUC, the IACUC of each institution and the IBC of SNU review 200–300 new protocols a year. In addition, the number of reviewers is approximately 10, except for one public institution specializing in animal testing.

**Table 2:** Status of protocol review in the Institutional Animal Care and Use Committees of various institutions

Type of institution	Number of		
	protocol review per year	Committee member (additional reviewer)	Administrative staff
Private company	25	8	0.5**
General hospital	240	10	1
Public institution	310	5	1
University A (national)	193	13	1
University B (private)	269	12	1
SNU-IACUC*	1000 (approx.)	15 (9)***	2
SNU-IBC* (for reference)	200	12	1

*SNU: Seoul National University; IACUC: Institutional Animal Care and Use Committee; IBC: Institutional Biosafty Committee

**One person in the company works as the staff of IACUC and IBC together

***In addition to fifteen official committee members, nine professional reviewers perform the protocol review in SNU-IACUC

These results show that in order to achieve a seamless protocol review while satisfying both the APA (which is currently limited to 15 members) and the LAA (which require a majority vote of all enrolled members for protocol approval), it is more realistic for SNU to operate at least three or more IACUCs. Although the restriction on the reviewer number in APA will be abolished soon, the majority vote of all enrolled members policy in the LAA remains unchanged. In the case of the IRB, SNU operates four committees in the Research affair, and the College of Medicine and the School of Dentistry each operate their own IRB.

#### Understanding of the process of animal protocol review

When performing animal experiments using human or transformed cells, approval from the IRB or the IBC and the IACUC is required. For smooth animal experiments, it is necessary to submit an animal protocol with prior approval from the IRB or the IBC in order for cells to be used. When the approval from multiple committees is required for an animal experiment, four out of six institutes (private company, general hospital, public institution, and SNU) required the pre-approval from the IRB and/or the IBC prior to protocol submission for review by the IACUC. In addition, the other two institutions (national university and private university) responded that all necessary committee approvals were required at the time of actual animal testing or experiments without any specific order.

In the US, when multiple committee approvals are required for the application of genetically modified organisms to humans or animals, there is no national-level guideline. However, each institution has its own guidelines. Some institutions require prior approval from the IBC for research involving animal experiment [12, 13], but conversely, there are cases where simultaneous or prior approval from the IRB or the IACUC is required during the IBC approval procedure [14]. Although there is no unified guideline, it is recommended that the committees of institutions maintain a close relationship and agree on procedures [15].

Table 3 presents the responses of the principal investigator, postdoc/graduate students, and reviewers as to whether researchers or reviewers are aware of the process when multiple committee approvals are required when writing or reviewing an animal protocol. When multiple committee deliberation was required, reviewers generally understood this and stated that the guidance of the institution was sufficient. However, over half of the principal investigators and postdoc/graduate students responded that they were unaware and did not receive adequate guidance from the institution. This suggests that researchers who write animal protocols require education about the content.

**Table 3:** Understanding of the process of animal protocol review when the protocol require approval by multiple committees

	Committee member (reviewer)	Principal investigator	Postdoc/student
Notified and understood	14 (73.7)	5 (33.3)	1 (8.3)
Notified but not understood	1 (5.3)	0 (0.0)	3 (25.0)
Not notified but understood	2 (10.5)	2 (13.3)	1 (8.3)
Not notified and not understood	2 (10.5)	8 (53.3)	7 (58.3)
Total	19 (100.0)	15 (100.0)	12 (100.0)

#### Institution’s supports and guarantee of autonomy to the IACUC

In the operation of the IACUC, support from institutions is important, but autonomy and independence from institutions are also essential [2]. In order to evaluate this, (1) institutional support, (2) a guarantee of independency, and (3) a reflection of the external committee members’ opinions were asked. The responses of the IACUC members are shown in Table 4. Approximately 70% of the IACUC members stated that the support and autonomy from the institution were secured, and over 80% of the members responded that the opinions of external members were well reflected.

**Table 4:** Institution’s supports and guarantee of autonomy to the IACUC

Items	Evaluation index		
	Supportive	Additional support required	
		Financial	Manpower (staff)
Support on IACUC operation	13 (68.4)	2 (10.5)	4 (21.1)

	Fully guaranteed	Partially guaranteed	Not guaranteed
Guarantee of autonomy	14 (73.7)	2 (10.5)	3 (15.8)

	Agree	Partially agree	Neutral	Partially disagree	Disagree	Refusal to answer
Reflection of the external committee members’ opinions	16 (84.2)	1 (5.3)	1 (5.3)	0 (0.0)	0 (0.0)	1 (5.3)

### Axial coding

Several noteworthy items were identified as requiring improvement in the operation of the IACUC through the survey. These are classified and summarized as follows.

#### Complaint to reviewers

When the protocol was not approved, the researchers were asked to write whether they agreed with the review results, if they disagreed, they were required to state the reason, and their complaints and suggestions about the protocol writing and review procedure. The researchers commonly highlighted the inconsistency of the review and complained that the reviewer failed to provide a valid rationale when requesting that the number of animals be reduced (Table 5). The former derives from the lack of the presentation of educational materials for the principal investigators and postdoc/graduate students who write the animal protocol, and the latter derives from the lack of education for reviewers.

**Table 5:** Responses of researchers when protocol review was not approved by the reviewer

List of reasons not to agree	Number of responses	
	PI (8)	PS (7)
After submission of amended protocol upon request, the reviewer pointed out other items not notified previously	3 (20.0)	1 (6.7)
Reviewers’ requesting reduction in the number of animals to use without reasonable evidence	2 (13.3)	3 (20.0)
Reviewers’ requesting to amend the previously approved protocol with no valid rationale	1 (6.7)	3 (20.0)
Others	2 (13.3)	0

Researchers’ complaints and requests about animal protocol preparation and review process	PI (15)	PS (12)
Providing researchers with good examples of various animal protocols along with specific feedback is required	6 (40.0)	6 (50.0)
Shortening approval delays is required	4 (26.7)	1 (8.3)
Intervening processes such as extension and repetition without any **o**ther protocol change need to be simplified	2 (13.3)	0 (0.0)
Amendment request from reviewers is hard to understand	1 (6.7)	3 (25.0)
Integrated management system for IRB, IBC and IACUC is required	2 (13.3)	2 (16.7)

*PI* principal investigator; *PS* postdoc/student

#### Case-oriented education with professional advice

The researchers basically accepted and respected the opinions and comments of the reviewers. However, as shown in Table 5, researchers emphasized the need for case-oriented education for researchers with professional advice. This opinion is also well expressed in Table 6, where many of the parties involved in animal experiments emphasized the education for reviewers and researchers.

**Table 6:** Main opinions and suggestions of participants to their institutions and government bodies about IACUC and animal experiments

To	Structured answers	CP	AD	FO	PI	PS
Institution	**Providing education for reviewers and researchers including online classes**	1	1	2		1
	Reducing the number of protocols to review	3				
	Reduction of protocol submissions through integrated review of similar protocols				2	
	Additional staff and/or financial support	1	1			
	Improving the notice method to researchers		1			1
	Strengthening post-approval monitoring activity			1		
	**Integrated management of IRB, IBC and IACUC**	1	1	4	2	1
	Standardization of checking in and out of animals			1	1	
	Providing consulting and technical support by professionals such as attending veterinarians				2	1
	Communication among IACUC, animal facility, and other related committees and institutions				1	1
Government	Legal guarantees of the independency and autonomy of the IACUC	2	1			
	IACUC regulatory amendments based upon the size of institution	1				
	Reinforcement of animal experiment alternatives through legal means	1			1	
	Minimum education requirement for reviewers and researchers		1	1		
	Mandatory hiring attending veterinarians		2	1		

Bold indicates the answers which were mostly and evenly distributed regardless of the respondents’ categories

*CP* committee member; *AD* administrator; *FO* facility operator; *PI* principal investigator; *PS* postdoc/student

#### Manpower support

In the open coding process, over 30% of the IACUC member respondents stated that the institution’s support was insufficient (Table 4). Regarding the lack of support for the IACUC, the demand for manpower support was predominantly high. This suggests that human resource support is more important than financial support in the operation of the committee. In addition, this is consistently shown in Table 6, which deals with recommendations to institutions and governments that emphasize human resource support such as attending veterinarians.

#### Opinions and suggestions by the respondents

The results were collected by allowing each survey respondent to freely describe their suggestions for their affiliated institutions and related government ministries. These are shown in Table 6. Suggestions for the provision or reinforcement of education for reviewers and researchers and requests for the integrated management of various committees were evenly distributed regardless of the respondents’ categories. As a request to the government, the IACUC members and administrators had opinions on legal guarantees of the independency and autonomy of the IACUC. In addition, in the case of administrators and animal facility operators, there were requests for educating IACUC reviewers and researchers and making it mandatory to hire attending veterinarians. Proposing these issues to the government rather than the affiliated institution, proves that the affiliated institution’s interest and support for those issues are limited.

Therefore, for seamless animal experiments through the improvement of the IACUC operation, we propose two topics: (1) reinforcing the education for researchers and reviewers, and (2) preparing an integrated management system for the relevant committees (IRB, IBC, and IACUC).

#### Reinforcement of the education for researchers and reviewers

Various forms of case-oriented education for researchers and reviewers are necessary. In particular, since the acceptance of online lectures by institutional members has increased, it is necessary to create online lectures of various cases at the national or institutional level and share the contents. Rather than complaining about the related regulations, researchers want education to develop their understanding of procedures and regulations to accept the relevant points. Therefore, education support that reflects this is required, and considering that it is difficult for each institution to bear this independently, support, such as the development of curriculum by the relevant government bodies, is required.

#### Preparation of an integrated management system for the relevant committees

Administrators and animal facility operators are complaining about difficulties in managing the APA, the LAA, the Bioethics and Safety Act, and the Living Modified Organism (LMO)-related laws simultaneously, so that researchers find out if there are any illegalities in the contents stipulated in multiple laws. Consequently, researchers must be aware of this and prepare for it. However, in reality, researchers are unaware of this. To make this possible, it is necessary to prepare an integrated management system for the relevant committees such as the IRB and IBC.

### Selective coding-storyline

This study categorizes key issues based on “Grounded Theory” by collecting opinions from researchers, reviewers, administrators, and facility operators on the operating conditions of animal experiments and the IACUC in institutions equipped with animal facilities. Based on these results, we compare the merits of each in the case of maintaining the current status and pursuing an effective step-by-step change. In addition, we suggest a desirable improvement plan for the operation of the IACUC and related organizations by analyzing the opinions from the respondents.

#### Maintaining the current status

If the current procedure is maintained, there is a high possibility of preparing an animal protocol in a situation where proper information is lacking, as highlighted by those involved in animal experiments. Animal protocols that require amendment are subject to judgment such as “conditionally approved with a request for revision” or “re-review after revision,” so the protocol applicant reflects the reviewer’s comments and understands their errors through the revision and gradually writes a high-quality animal protocol. However, researchers believe that most of the items highlighted by reviewers (errors or mistakes in writing) can be avoided if there is prior education and training, which can shorten the time from submission to approval.

Researchers are often dissatisfied with the reviewer’s review criteria, mainly because the reviewers make new comments about the protocol approved by other reviewers in the past and reject it. Not all IACUC members who conduct reviews are experts in animal experiments; therefore, it is important to educate reviewers to enable consistent reviews. In addition, the laws related to animal experiments (APA and LAA) are also being periodically revised, and even if the administrator of each institution is notified of this, the relevant information cannot be delivered to researchers in a timely manner or reviewers cannot recognize it. Therefore, it cannot be reflected in the protocol review. Regarding these legal changes, each institution should periodically educate reviewers and researchers in order to avoid future difficulties.

#### Attempt to partial or total change

The results of this study indicate that changes are inevitable to improve the management of animal experiment procedures based on the IACUC, including ethical perspective. Above all, various forms of practical education are necessary for all participants related to animal experiments, including researchers and reviewers (IACUC members). Consequently, three suggestions have been proposed in this study: (1) education for researchers on how to write a protocol according to the type of animal experiment, (2) standardization of screening criteria for various animal experiments by presenting various cases for the IACUC members who review the protocol, and (3) training on a detailed understanding of relevant laws and policies for administrators and animal facility operators. The reasons for each proposal are as follows.

1. *Education for researchers on how to write a protocol according to the type of animal experiment* As experimental techniques using animals are diverse and advanced, prerequisites for planning are increasing, and there are cases in which researchers fail to prepare a complete protocol because they are not well aware of it. If education in this regard is effective, researchers can easily determine what type of animal experiment is included by writing the protocol according to the research purpose. Therefore, unnecessary re-review will be reduced.
2. *Standardization of screening criteria for various animal experiments by presenting various cases for the IACUC members who review the protocol* Since not all IACUC members who review the protocol are experts in the relevant animal experiments, it is occasionally difficult to decide what kind of decision suits each type of experiment. Each member has a variety of perspectives depending on their position (e.g., animal researchers, animal activists, religious people, lawyers, and researchers on alternative methods of animal testing). In addition, there may be differences of opinion between members with long-term review experience and new members. However, from the researcher’s perspective, since the least consistent decision is expected for the same animal experiment, each institution regularly educates reviewers using typical cases for each type of experiment as an example based on the protocol deliberation results previously accumulated. Consequently, it is necessary to help those who review the criteria to make a decision in a state of mutual agreement on some basic matters.
3. *Training on a detailed understanding of relevant laws and policies for administrators and animal facility operators* Administrators and facility operators are in the position of receiving the most complaints from researchers. However, are also obliged to deliver the most recent and relevant information to researchers. In accordance with changes in society’s perception of companion animals, related laws are also periodically strengthened or revised; therefore, each institution needs to frequent communicate this to researchers. In addition, it is necessary to support them to receive the education provided by experts, or to attend public hearings on government policies and events of related societies and civil society organizations.

**Fig. 1:**
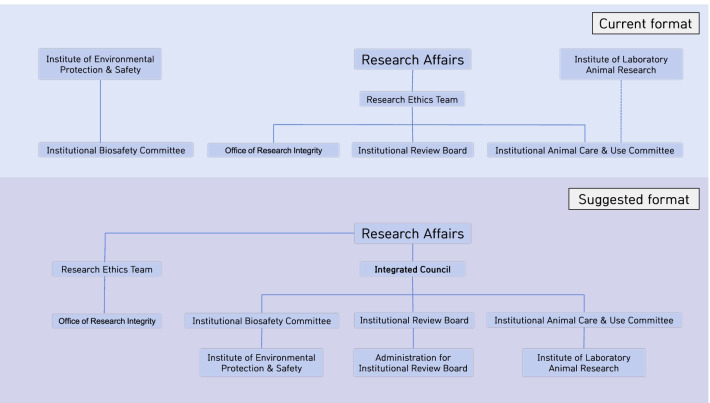
A schematic diagram of an integrated management system for Seoul National University

In addition to providing various forms of education, taking SNU as an example, we propose an integrated management system improvement plan for the relevant committees as shown in the schematic diagram below (Fig. 1). As a result of the survey and online search, there are many cases where the IACUC and the IBC are jointly operated and managed in Korea and international cases. However, in the case of SNU, as described in Fig. 1, the Research ethics team is in charge of administration of the IRB and the IACUC, while ILAR manages technical support and facility management related to animal experiments separately. However, for the IBC, the Institute of Environmental Protection & Safety (IEPS) provides administrative and technical support.

However, as research using genetically modified organisms or translational research becomes active, the need for information sharing between the IBC, the IACUC, and the IRB increases and researchers’ demands for an integrated organization to seamlessly manage them are also increasing. Therefore, the Research ethics team is continuously focusing on the Office of Research Integrity, which involves its original tasks such as publication ethics and conflict of interest in research and separately forms an integrated management organization or consultative body that manages mutual communication by overseeing the IRB, the IACUC, and the IBC. Furthermore, administrative and educational support for each committee will be provided by the IEPS for the IBC and by the ILAR, which is currently unconnected with the IACUC, for the IACUC. In the case of the IRB, it is proposed that the administrative and education support team will manage a total of six subcommittees (1st, 2nd, 3rd, 4th, Medical, and Dental), which are separately operated by Research Affairs, as depicted in Fig. 1. Unlike SNU, in a small institution, administrative and educational support can be operated by an integrated administrative team.

## Discussion

It will be difficult for each individual institution to conduct all these trainings independently. As with the public IRB education program operated by the National institute for Bioethics Policy [16] and the research safety education program of the National Research Safety Headquarters [17], it is necessary to consider operating a public IACUC. This manages the IACUC of each institution and supports education for administrators, animal facility operators, researchers and IACUC members who review animal protocols, along with various curriculum developments by government-related ministries, directly or through related organizations. Currently, in Korea, the Korean Association for IACUC [18] and the Bioethics Information Center [19] are providing the IACUC education programs, and some institutions are providing their own education programs. However, there are many differences in the scale, content, and scope of education. In the US the majority of institutions use the Collaborative Institutional Training Initiative (CITI) Program [20] to provide training programs for researchers and reviewers on the IRB, the IACUC, and the IBC. Therefore, the establishment of an integrated educational support organization in Korea may be considered in the future. For reference, in Korea, the US CITI program is provided in connection with BIC Study.

The IRB, which reviews human research, and the IBC, which reviews the use of cells and tissues and LMOs, are managed and supervised by a government department different from the IACUC. As animal experiments become increasingly sophisticated, studies that require approval from both the IACUC and the relevant committee are increasing. Consequently, rather than prescribing prerequisites for the deliberation of a specific committee, it is desirable to create an integrated management body for the relevant committees within the institution that effectively communicate when researchers conduct animal experiments. This will ensure that the animal facility operator or members of each committee conducting a review can more easily verify the relevant protocols with each other. Such a system can support researchers to conduct more ethical and rational animal experiments while complying with the relevant laws. In addition, the integrated management body must focus on the education of the researchers. Many institutions in the US have various educational service programs in their division or office of research compliance [21, 22]. Therefore, the government should also check whether there are any contradictions between the relevant regulations of each committee. If certain areas require change, they should be actively improved from a researcher-centered perspective.

## Conclusions

The results of the surveys suggest that institutions that conduct animal experiments must continuously provide education related to their duties for researchers and the IACUC members. In addition, they must establish a unified/integrated management organization or consultative body with relevant committees such as the IRB and the IBC. If greater levels of education and an integrated management system are established, it will be possible to enhance the excellence of researchers and to better manage the operation of the IACUC.

## Methods

The methodological framework consists of a qualitative approach. The study was conducted with the IRB approval from the SNU School of Dentistry (IRB approval number: S-D20210022). The survey was conducted by email and the participants provided informed consent.

### Theoretical sampling of respondents

Theoretical sampling is purposeful sampling according to categories that we developed and these categories are based on theoretical concerns. Sixty people were surveyed through purposeful sampling. In the case of the SNU researchers, the main respondents, the target principal investigators, and graduate students/postdocs were selected to reflect the representativeness of each campus and major colleges. In addition, animal facility operators, administrators, and the IACUC members of each institution were selected as survey subjects from institutions that model IACUC according to the type of institution such as private company, university, and hospital.

SNU conducts 5% of Korea’s total animal protocol reviews and also uses 10% of the national laboratory animal usage. It is a vast research institute that consumes approximately 350,000 experimental animals per year, mainly rodents, at 15 official animal facilities located on four campuses [2]. This is close to the total annual use of laboratory animals in the Netherlands or Israel [23]. In addition, being a university, the types of animal experiments performed and the affiliation of researchers vary and it is possible to collect a variety of samples and cases—unlike institutions that only conduct research on specific animal species or specific fields.

Therefore, the questionnaire was designed for the principal investigators and researchers who submit animal protocols which will be reviewed by the IACUC and the animal facility operators who manage the laboratory animals. However, for the analyses related to the operation of the IACUC, comparative analyses must be performed according to the type and size of institution including university, public institution, and private company. Therefore, in the case of the IACUC members and administrators, a survey was conducted targeting the IACUC members and administrators in universities (national and private), public institutions, and private companies that operate the IACUC in Korea.

### Questionnaire composition of the survey

The questionnaire was composed of items necessary for improving the operation of IACUC based on the opinions of experts in the field, such as animal facility operators and administrative personnel, referring to various suggestions of SNU animal researchers posted over the past several years. The survey targets were classified as the IACUC members, administrators, animal facility operators, principal investigators, and researchers (postdocs and graduate students). In addition, the questionnaire contents were divided into the IACUC members, administrators, animal facility operators, and research personnel (principal investigators, postdocs and graduate students) according to their duties.

### Data analyses

The survey was conducted and analyzed as follows. First, similar answers were grouped in the data, categorized, and open coding was performed to suggest response rates for each item. Subsequently, items (topics) showing significant results were extracted, and among them, the key issues necessary for operational improvement of the IACUC were selected and survey results were derived through an axial coding process. Subsequently, the study was conducted through the process of searching for alternatives through selective coding, which analyzes the questionnaires asking about differences in opinions by groups.

## Data Availability

Data of the study may be available upon reasonable request to the corresponding authors.

## Abbreviations

APA: Animal Protection Act
CITI: Collaborative Institutional Training Initiative
IACUC: Institutional Animal Care and Use Committee
IBC: Institutional Biosafety Committee
IRB: Institutional Review Board
LAA: Laboratory Animal Act
LMO: Living Modified Organism
SNU: Seoul National University
